# A multi-objective sequence engineering framework for rational genetic vaccine design

**DOI:** 10.1098/rsif.2026.0525

**Published:** 2026-10-21

**Authors:** Je Hun Moon, Seo-Young Park, Kok Siong Ang, Yoon Ki Kim, Dong-Yup Lee

**Affiliations:** ^1^ School of Chemical Engineering, Sungkyunkwan University, Suwon, Republic of Korea; ^2^ Centre for Biomedical Data Science, Duke-NUS Medical School, Singapore, Singapore; ^3^ Department of Biological Sciences, KAIST, Daejeon, Republic of Korea

**Keywords:** COVID-19 vaccine design, codon optimization, tissue specificity, CpG dinucleotide, motif screening

## Abstract

Genetic vaccine performance is determined not only by the delivery platforms and antigen choice but also by the coding sequence that influences antigen expression, innate immune sensing, transcript stability and sequence-associated risks. However, the design principles governing these sequence-level effects remain insufficiently defined. Thus, we consider vaccine coding-sequence design as a multi-objective engineering problem that requires simultaneous consideration of multiple, potentially competing biological properties. We present a multi-parameter computational framework for analysing SARS-CoV-2 vaccine coding sequences, including BNT162b2 (Pfizer-BioNTech), mRNA-1273 (Moderna), AZD1222 (AstraZeneca) and several academic vaccine candidates. The framework evaluates codon usage, codon-pair context, CpG content, regulatory motifs, GC content and localized sequence-structural features within a unified quantitative design space. Our analysis shows that existing vaccines use diverse design strategies and that no single metric adequately captures their design complexity. By enabling systematic comparison across multiple parameters, the framework supports the rational generation and prioritization of candidate sequences with defined biological trade-offs. This work bridges computational sequence analysis, vaccine biology and engineering design, providing a foundation for context-aware optimization of genetic vaccine sequences across delivery platforms, target tissues and emerging pathogens.

## 1. Introduction

The COVID-19 pandemic has highlighted an important challenge at the interface of computational sequence science and molecular vaccinology. A wide range of vaccine platforms, including inactivated-virus, live attenuated, recombinant protein, adenoviral vector, influenza virus vector, DNA and mRNA vaccines, have demonstrated substantial clinical utility. Among them, genetic vaccines, particularly mRNA and viral vector-based platforms, have gained prominence because of their high efficacy, scalability and adaptability [1,2]. Although both can encode the same antigen, they differ substantially in intracellular processing. mRNA vaccines are translated directly in the cytoplasm and are therefore influenced by RNA degradation and innate immune sensing, whereas viral vectors deliver DNA to the nucleus, where antigen expression requires transcription and is subject to host regulatory processes [3]. Despite these mechanistic differences, vaccine development has largely focused on delivery systems and immunogenicity, while systematic optimization of antigen-coding sequences has received comparatively limited attention.

In particular, effective genetic vaccine design requires the selection of synonymous codons that improve antigen expression while preserving the encoded amino acid sequence and minimizing sequence-associated liabilities [4]. This process, commonly referred to as codon optimization, is inherently multi-factorial, as tissue-specific codon usage, CpG dinucleotide abundance and distribution, regulatory motifs and potential off-target risks may create competing effects on translation, stability and innate immune sensing. Codon usage can affect expression across cell and tissue types, which is relevant because vaccine-derived nucleic acids and their delivery vehicles may distribute outside the intended target cells [5–8]. CpG dinucleotides also require careful consideration, as their effects on transcript stability, immune recognition and sequence performance vary according to the vaccine platform, CpG abundance and local sequence context [9–11]. These factors are individually important, but they have not yet been systematically integrated into a unified framework for vaccine sequence design. This limitation is particularly relevant given the substantial variation in the clinical performance of COVID-19 vaccines. For example, BNT162b2 (Pfizer-BioNTech) and mRNA-1273 (Moderna) achieved high efficacy, whereas CureVac's CVnCoV showed substantially lower efficacy despite using a broadly similar delivery technology [12,13]. Similarly, the viral vector vaccine AZD1222 elicited lower antibody titres than the mRNA vaccines [14]. Although such differences arise from multiple platform- and product-specific factors, they provide a clear rationale for a methodology to assess how coding-sequence design may contribute to vaccine performance.

Here we present a multi-parameter computational framework for evaluating vaccine coding-sequence design, with a focus on codon optimization and related sequence features. Using clinically used and academic SARS-CoV-2 vaccine sequences as a real-world reference set, we first compare the design strategies of BNT162b2, mRNA-1273, AZD1222 and additional vaccine candidates to identify distinct design choices and sequence-level trade-offs. We then use these findings to formulate a systematic framework that integrates tissue-specific codon usage, codon-pair context, regulatory sequence motifs and CpG composition as factors potentially affecting antigen expression and sequence-associated innate immune sensing.

## 2. Results

### 2.1. Global comparative landscape of vaccine coding sequences

To compare sequences of COVID-19 vaccines (supplementary material, table S1), we collected sequence information for mRNA vaccines BNT162b2 and mRNA-1273 [15], the adenoviral vector-based vaccine AZD1222 [16,17], and the reference SARS-CoV-2 spike protein from NCBI (NC_045512.2). We also included four mRNA vaccine candidates (LD1, LD2, OG and mRid) [18,19], designed using codon optimization tools, including LinearDesign [18], OptimumGene (GenScript) and mRNAid [19]. In BNT162b2 and mRNA-1273, two amino acids at the apex of the central helix within the S2 subunit core (K986P and V987P) are replaced with prolines to stabilize the spike protein by preventing prefusion-to-postfusion conformational changes [20]. Multiple stop codons are also incorporated: BNT162b2 contains two consecutive UGA stop codons, whereas mRNA-1273 employs all three stop codon variants (UGA-UAA-UAG). This redundancy aims to reduce ribosome read through, which can otherwise produce translation of 3′ untranslated regions and unintended C-terminal amino acid additions [21]. AZD1222 retains the same amino acid sequence as the wild-type (WT) spike protein but is preceded by a tissue plasminogen activator (tPA) signal peptide; for direct comparison, we replaced this leader with a start codon.

As expected, the vaccine sequences were markedly dissimilar to the WT, with pairwise codon-alignment similarity scores of no more than 30% (Material and methods; figure 1A). However, similarity among the vaccine sequences ranged from 47 to 95%. BNT162b2 and AZD1222 were the closest, with approximately 95% similarity and variation at only 68 codon positions. These differences comprised 66 synonymous changes and the K986P/V987P amino acid substitutions present in BNT162b2. Despite this high sequence similarity, their reported efficacies against symptomatic COVID-19 were substantially different: 95.0% for BNT162b2 and 66.7% for AZD1222 [13]. This disparity indicates that codon-level similarity alone does not determine product-level efficacy, which is also influenced by antigen design, delivery platform and other product-specific factors [22]. A comparable dissociation is evident for mRid, which shares 91.0% codon similarity with mRNA-1273 (reported efficacy of 94.1% [13]), but produces lower spike-protein expression [19].

**Figure 1.**
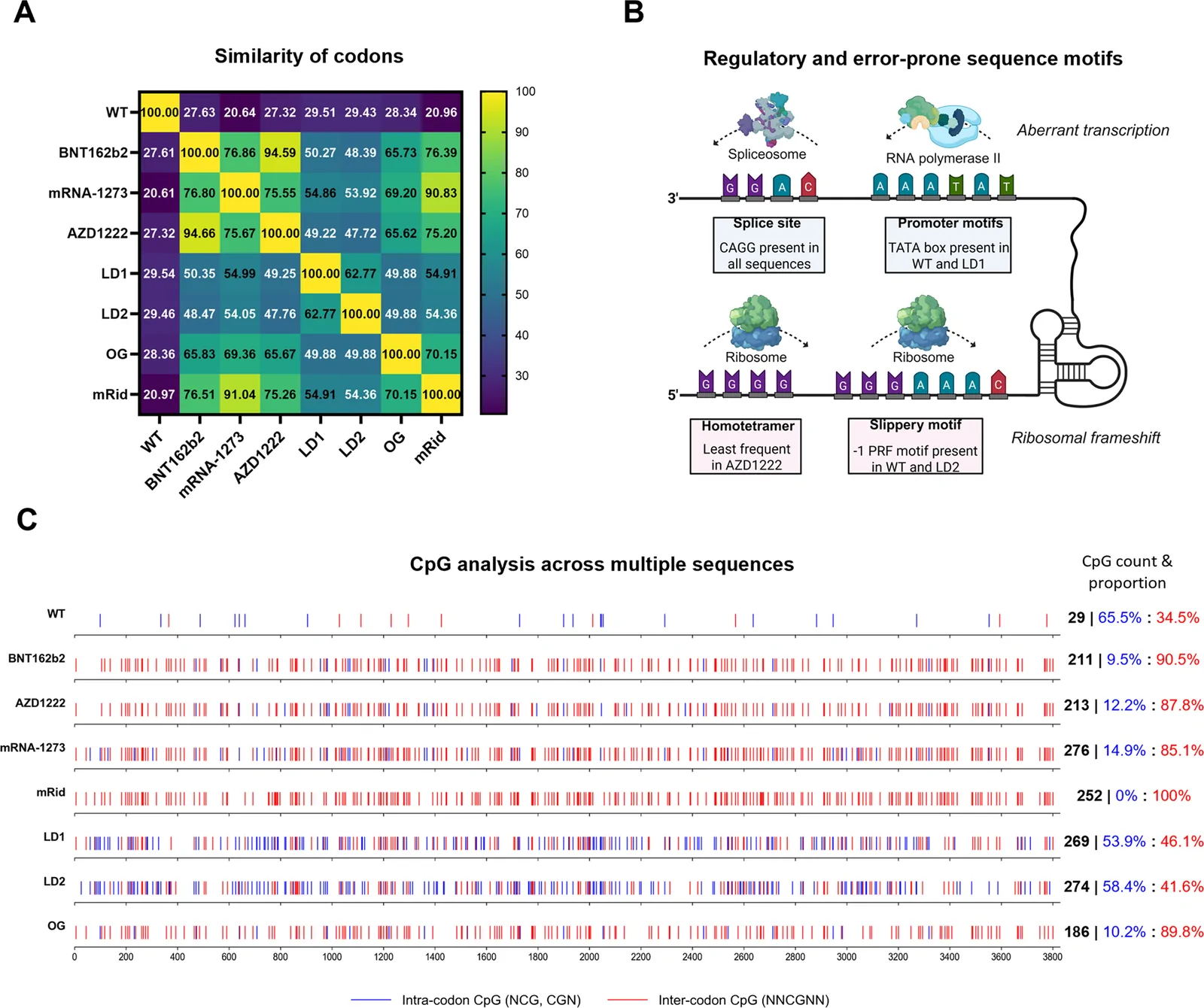
Comparison of WT, clinically used vaccine sequences and vaccine candidates. (A) Pairwise codon sequence similarity (%). WT shows <30% similarity to vaccine sequences, whereas clinically used vaccines share high similarity (≥75%); LD1 and LD2 show approximately 50% similarity to other sequences, and BNT162b2 and AZD1222 display the highest pairwise similarity. (B) Detected regulatory motifs, including splice sites, core promoter elements, homotetramers and slippery motifs (associated with aberrant transcription and ribosomal frameshifting). Motifs identified within each sequence are summarized in the corresponding boxes. (C) CpG site distribution across sequences with intra-codon CpGs in blue and inter-codon CpGs in red, ordered by codon similarity for ease of comparison. Inter-codon CpGs predominate in all sequences except WT, LD1 and LD2. Panel (B) was created using BioRender.com.

The sequences were next screened for functional motifs (table 1), defined here as short, recurrent elements with established biological roles and characteristic positional contexts. The initial analysis focused on potentially unfavourable regulatory motifs whose ectopic occurrence within coding regions could disrupt gene expression or protein production (figure 1B). TATA and CAAT boxes normally function as promoter-associated elements upstream of transcription start sites; however, their presence within coding regions may promote unintended transcription initiation by aberrantly recruiting the transcriptional machinery [23–25]. Similarly, cryptic splice sites may induce aberrant RNA processing and generate truncated or otherwise altered transcripts [26,27]. The screening included TATA (TATAAA) and CAAT (GGYCAATCT, where Y = C or T) boxes [28], as well as canonical eukaryotic splice sites (3′ acceptor sites CAGG and 5′ donor sites MAGGTRAGT, where M = A or C and R = A or G) [29]. We identified one TATA box in LD1 and two in the WT, suggesting potential cryptic promoters for alternative transcription initiation (ATI) [30]. Although ATI can facilitate viral adaptation and enhance viral fitness [31,32], such elements may be undesirable in vaccine sequences unless intentionally designed.

**Table 1.** Summary of length, number of exclusion motifs, slippery motifs, homotetramers, GC content, number of CpG motifs and remarks across WT, vaccine sequences and vaccine candidate sequences for COVID-19.

	length (nucleotide)	exclusion motif (count)	slippery motif (–1 frame)	homotetramers (count)	GC content (%)	CpG motif (count)	remarks
WT	3822	CAGG (18), TATAAA (2)	TTTAAAC	AAAA (26), CCCC (1), GGGG (1), TTTT (39)	37.31	29	—
BNT162b2	3825	CAGG (15)	—	AAAA (2), CCCC (21), GGGG (3), TTTT (1)	56.97	211	UGA-UGA stop codon, proline substitution
mRNA-1273	3828	CAGG (17)	—	CCCC (32), GGGG (2)	62.23	276	UGA-UAA-UAG stop codon, proline substitution
AZD1222	3822	CAGG (14)	—	AAAA (2), TTTT (1)	56.59	213	UGA stop codon, no proline substitution, tPA leader replaced with ATG for analysis
LD1	3819	CAGG (17), TATAAA (1)	—	AAAA (1), CCCC (18), GGGG (20), TTTT (2)	54.83	269	no stop codon, no proline substitution
LD2	3819	CAGG (16)	GGGAAAC	CCCC (9), GGGG (21), TTTT (5)	55.30	274	no stop codon, no proline substitution
OG	3819	CAGG (35)	—	CCCC (10), GGGG (2), TTTT (6)	55.04	186	no stop codon, no proline substitution
mRid	3819	CAGG (37)	—	CCCC (35), GGGG (3)	62.32	252	no stop codon, proline substitution

### 2.2. Hidden error-prone elements and CpG motif profiling

The next analysis examined nucleotide motifs with defined sequence patterns but no fixed positional constraints, allowing their cryptic occurrence throughout coding regions and potentially increasing transcriptional or translational error rates. Slippery-site motifs associated with ribosomal frameshifting were first assessed (table 1) [33]. The WT sequence contained the TTTAAAC motif at position 2874, with the start codon defined as position 1. This motif is a well-characterized trigger of −1 programmed ribosomal frameshifting (−1 PRF) in coronaviruses. LD2 contained the GGGAAAC motif at position 549, which was also reported to induce −1 PRF in higher eukaryotes. The presence of such motifs may have biological significance because a recent study linked N1-methylpseudouridine (m¹Ψ) to +1 ribosomal frameshifting in mRNA vaccine contexts and detected T-cell responses against frameshift-derived products in both mice and humans [34]. Although this mechanism differs from −1 PRF, it demonstrates that translational frameshifting can generate products with immunological consequences. In practice, slippery motifs generally require additional stimulatory structures, such as downstream pseudoknots, to promote frameshifting [33]. This issue may be especially important for LD2, as its design minimized predicted folding free energy and may therefore favour stable RNA secondary structures. Homotetramers were also examined as potential error-prone elements. These were defined as maximal contiguous runs of four or more identical nucleotides, with each run counted once rather than through overlapping 4-nt windows. Such simple sequence repeats are susceptible to polymerase slippage during replication or transcription [35,36]. AZD1222 contained only three homotetramers compared with 18 in OG, the sequence with the second-lowest count. Since adenoviral vectors undergo nuclear transcription and may generate spliced transcripts, whereas mRNA vaccines function in the cytoplasm, minimizing homotetramers in AZD1222 may theoretically reduce transcriptional slippage. These findings support systematic identification and mitigation of cryptic and error-prone elements in vaccine sequence design.

Balancing sequence-associated immune sensing with expression-related design objectives is a central challenge in genetic vaccine design. CpG dinucleotides are particularly relevant because unmethylated CpG motifs are common in bacterial and viral DNA but relatively underrepresented in vertebrate genomes. In DNA-based platforms, these motifs can activate Toll-like receptor 9 (TLR9) [37], whereas CpG elements in mRNA are recognized by zinc finger antiviral protein (ZAP) [38]. CpG profiling identified only 29 dinucleotides in the WT sequence, consistent with the CpG depletion characteristic of the SARS-CoV-2 lineage [39]. By contrast, all vaccine sequences contained substantially more CpGs. mRNA-1273 had the highest count at 276, while the codon-matched mRid sequence contained 252 CpGs. BNT162b2 and AZD1222 carried nearly identical numbers with 211 and 213 CpGs, respectively. LD1 and LD2 each harboured approximately 270 CpGs, despite their markedly distinct codon-usage profiles.

Beyond simple enumeration, CpG formation must be examined in terms of both positional distribution and local sequence context, as these features can influence its immunological effects. Codon composition was therefore analysed for each sequence (supplementary material, table S2); CpGs were classified as either intra-codon or inter-codon dinucleotides (figure 1C). Intra-codon CpGs occur in CGN codons (where N denotes any nucleotide) encoding arginine or in NCG codons encoding alanine (GCG), proline (CCG), serine (TCG), or threonine (ACG). Inter-codon CpGs arise when a codon ending in C is immediately followed by a codon beginning in G. Genetic-code degeneracy allows C-ending codons to encode 15 amino acids, whereas G-starting codons encode only five: valine, alanine, aspartate, glutamate and glycine. Among the analysed sequences, mRid contained the largest number of C-ending codons, with 825 accounting for 64.8% of all codons. Its highly restrictive codon adaptation index (CAI)-based design, which assigns a single preferred codon to each amino acid, generated CpGs exclusively across codon boundaries, with no CpG occurring within a single codon. In the clinically used vaccine sequences and OG, 85–91% of CpGs likewise originated at codon boundaries. By contrast, the corresponding proportions were substantially lower in LD1 and LD2, at 46.1 and 41.6%, respectively. These contrasting patterns indicate that similar CpG abundance can arise from markedly different codon compositions and local sequence contexts, supporting a multi-parameter approach that jointly considers CpG distribution, sequence motifs and codon usage in vaccine sequence optimization.

### 2.3. Systematic framework for vaccine sequence design

Codon optimization is a computational strategy that enhances transgene expression by substituting rare codons or codon pairs with synonymous codons that exhibit higher usage frequency in the target host organism or tissue. In the context of vaccine development, codon optimization should be tailored not only to support antigen translation but also to manage sequence-linked features that may influence innate sensing and transcript behaviour. To address these multi-factorial objectives, we present a systematic framework that integrates multiple evidence-based design criteria and quantitative sequence parameters to generate candidate coding sequences for prioritization (figure 2). This framework requires two primary inputs: the amino acid sequence of the target antigen and a codon reference bias specific to the tissue of interest.

**Figure 2.**
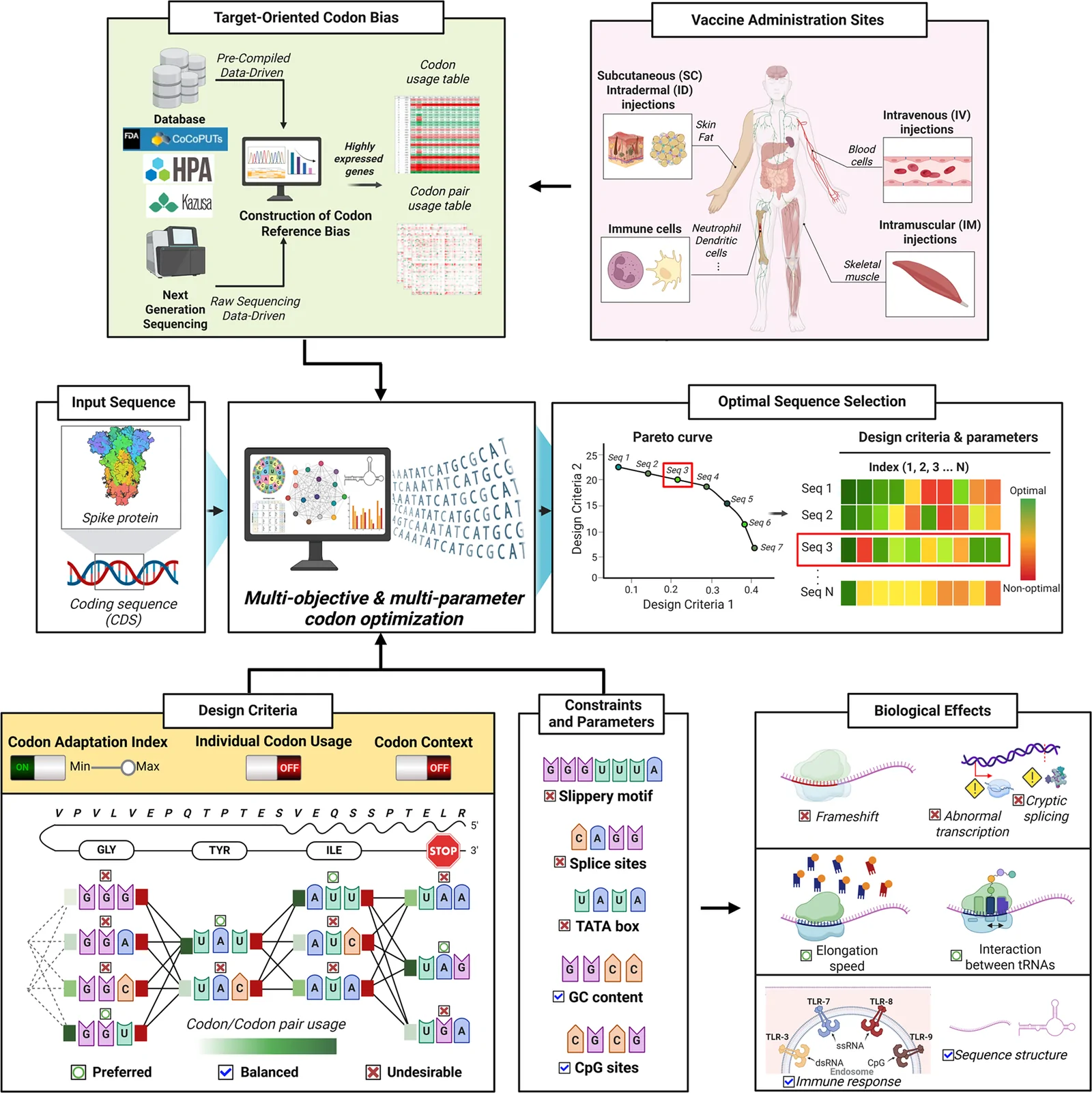
Systematic framework for designing vaccine sequences. The framework integrates multiple inputs and optimization steps to generate optimal vaccine sequences. Inputs include the target antigen gene and a target-oriented codon bias derived from pre-compiled databases or omics data (commonly transcriptomic data focused on highly expressed transcripts to capture the target tissue's translational environment). Codon usage aligned with the desired host or target tissue can enhance protein expression and stability. Inputs are processed through a multi-objective optimization that adjusts codon indices for translational efficiency while modulating sequence parameters (CpG sites affecting immune responses and GC content affecting mRNA structure). Undesirable motifs are excluded as design constraints. Candidate sequences are iteratively evaluated based on whether their biological features are preferred, balanced or undesirable, and the final selected candidate is highlighted in red. Figure created using https://www.biorender.com/.

A tissue- or cell-type-specific codon reference can better represent the cellular environment encountered by an administered genetic vaccine. The route of administration influences the cell populations initially exposed to the vaccine and the surrounding immune and translational milieu, although vaccine components may also distribute beyond the injection site. Context-specific codon-usage profiles can be obtained from curated databases or constructed using high-throughput omics datasets. Transcriptomic data are commonly used because of their abundance and accessibility, whereas ribosome-profiling and proteomic data can provide more direct information on translational activity. An expression-weighted codon reference can be constructed by selecting highly expressed transcripts from relevant tissues or cell types under defined biological and clinical conditions, including age, sex, genetic background, health status and pre-existing immunity [40–42]. Such a reference captures codon and codon-pair patterns enriched among genes actively expressed in the biological context of interest.

The next step is to define the design criteria, adjustable sequence parameters and hard biological constraints. The principal criteria considered here include CAI, individual codon usage (ICU) and codon context (CC). CAI quantifies the extent to which a sequence favours frequently used synonymous codons in the selected reference [43], whereas ICU and CC provide complementary information on individual-codon and codon-pair preferences [44], respectively. In particular, CC captures host-associated codon-pair patterns that may influence translational elongation and ribosome dynamics. Adjustable parameters include GC content and CpG dinucleotide frequency. GC content can affect RNA structure and stability, whereas the consequences of CpG abundance and distribution depend on the vaccine platform and cellular context. Platform-relevant constraints are also applied to remove potentially unfavourable features, including repetitive sequences and regulatory motifs associated with frameshifting, unintended transcription initiation or cryptic splicing.

Given the target antigen and the context-specific codon reference, candidate coding sequences are optimized according to the selected criteria, parameters and constraints (Material and methods). Multi-objective optimization generates a Pareto front of non-dominated solutions, each representing a distinct compromise among competing criteria, such as CAI, ICU and CC. This approach avoids defining a single universally optimal sequence and instead provides a set of candidates with quantitatively characterized design trade-offs.

### 2.4. Codon optimization of COVID-19 vaccine sequences

To demonstrate the applicability of the framework, codon optimization was performed under two principal settings: single-objective optimization based on CAI and multi-objective optimization combining ICU and CC criteria. The CAI-based setting was further divided into an unconstrained case, in which CAI was the sole objective, and a constrained case incorporating additional sequence parameters and biological restrictions. Skeletal muscle was chosen as the target site owing to its relevance in vaccine delivery [13] and its crucial role in upregulating the expression of cytokines, chemokines and cell adhesion molecules [45] (Material and methods).

The unconstrained CAI optimization generated 441 sequences that reached the maximum CAI value of 0.999 (supplementary material, table S3). These sequences had a mean GC content of 58.5% and contained 220–222 CpG dinucleotides, values comparable to those of BNT162b2 and AZD1222, whose CAI values were 0.953 and 0.950, respectively. mRid, which was optimized for CAI using a human codon-usage profile [19], also exhibited a high skeletal-muscle CAI of 0.976 but had distinct GC and CpG compositions (table 1; supplementary material, table S4). This comparison illustrates that similarly high CAI values can arise from sequences with substantially different compositional properties.

The mean GC content of the CAI-optimized sequences was close to the reported average for human coding sequences (52.3 ± 8.5%) [46] and remained within the range of 30–70% [47]. Although increased GC content is often associated with greater mRNA stability and expression [48], this relationship is not universal. GC-rich regions can promote stable secondary structures, whereas G-rich regions can form G-quadruplexes that may impede transcription or translation [49,50]. A GC-content constraint of 30–70% was therefore applied to avoid extreme sequence compositions. For CpG content, the expected CpG frequency of 1/16 in bacterial and viral DNA [51] corresponded to approximately 239 CpGs for the analysed coding-sequence length. Note that this value was used as a quantitative design reference rather than as a universal biological optimum. Candidate cryptic promoter and splice-site motifs were also excluded as sequence-level constraints. Among the sequences satisfying all constraints, the highest CAI was 0.897, lower than that obtained through unconstrained CAI optimization. The constrained design avoided the candidate 3′ splice-site motif CAGG detected in the CAI-only sequence set. Although the presence of this short motif alone does not establish splice activity, its removal reduces a potential sequence-associated risk in contexts permissive to aberrant RNA processing [27,52].

Multi-objective optimization was subsequently performed using ICU and CC under the same parameter ranges and biological constraints. Whereas CAI favours codons with high individual weights in the selected reference, ICU and CC preserve broader host-associated patterns of codon and codon-pair usage [44]. ICU-based optimization limits excessive concentration on a restricted set of preferred codons, while CC accounts for codon-pair organization that may influence elongation dynamics and local ribosome behaviour [53,54]. CpG abundance was systematically varied across 12 design scenarios, comprising 11 CpG targets from 0 to 290 in increments of 29, based on the WT count of 29 CpGs, and one unconstrained case. The biological consequences of CpG content are platform- and context-dependent. In DNA vaccines and viral-vector genomes, limited CpG enrichment may enhance cytokine production and adaptive immune responses [55], whereas excessive CpG content can activate TLR9 and, under some conditions, contribute to systemic inflammatory responses [56,57]. In mRNA vaccines, CpG-enriched RNA can instead be targeted by ZAP [58]. Innate responses to ionizable lipid nanoparticles can promote type-I interferon signalling [59], and ZAP is itself an interferon-stimulated gene [60]. Excessive CpG enrichment may consequently reduce mRNA stability or translation in some cellular contexts [61]. One representative sequence was selected from the Pareto front generated under each design scenario for subsequent analysis (supplementary material, table S1).

The optimized sequences attained higher ICU and CC values than the comparator vaccine sequences (figure 3A). To retain sequence diversity rather than converge on closely related solutions, representative candidates were selected with low similarity to one another and to the comparator sequences (figure 3B). The ICU–CC-optimized sequences had a mean pairwise similarity of 43.6%, while their mean similarity to the comparator sequences was 42.2%. As the CpG target increased, the proportion of CpGs formed across codon boundaries also increased before gradually reaching a plateau (figure 3C), indicating a nonlinear relationship between CpG abundance and codon-pair composition. Collectively, these results demonstrate that favourable ICU and CC values can be maintained across different CpG constraints and illustrate the need to consider interacting sequence objectives when prioritizing vaccine candidates.

**Figure 3.**
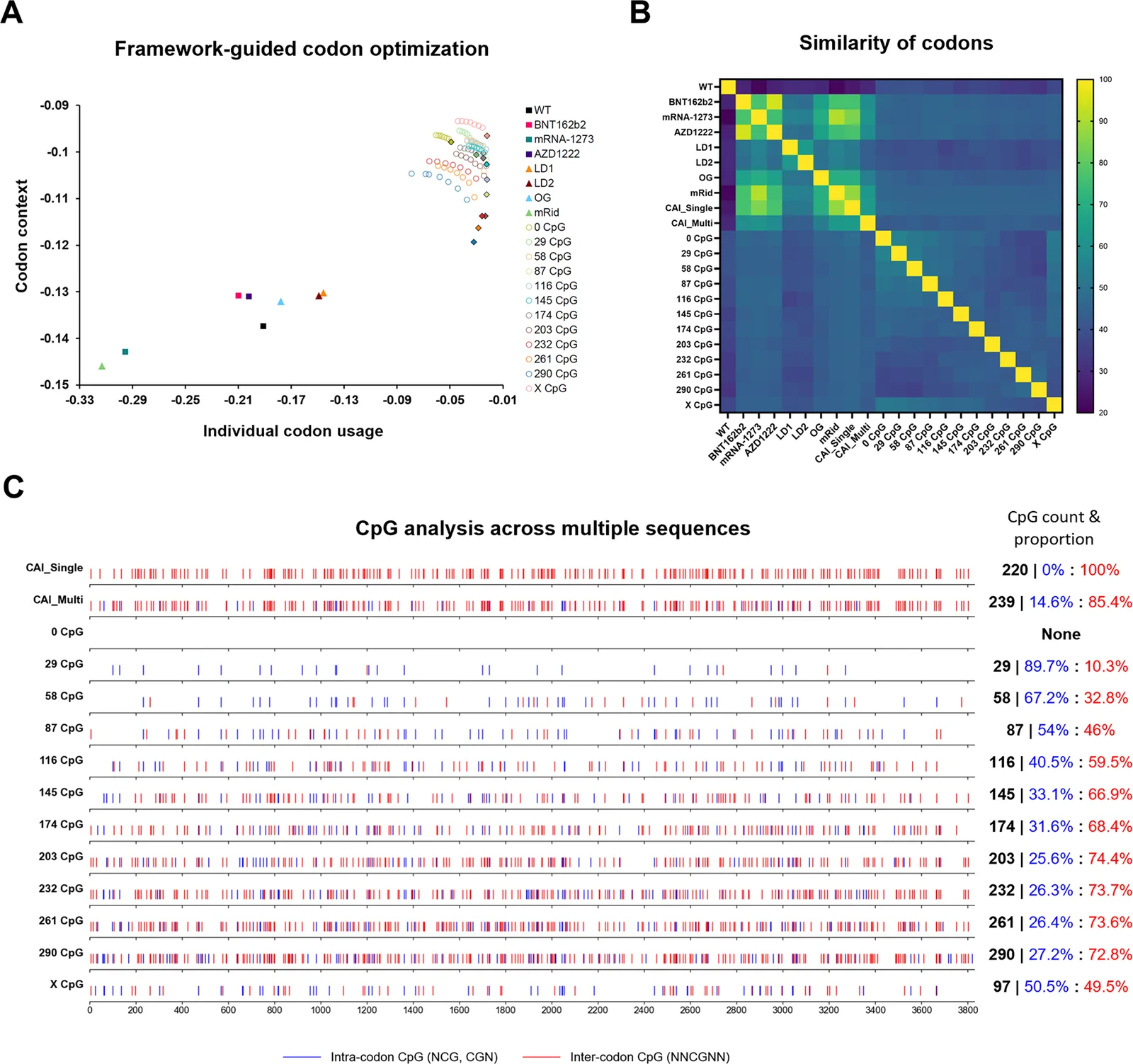
Results of multi-objective codon optimization using the proposed framework. (A) Pareto-optimal sequences generated by multi-objective optimization on ICU and CC fitness with GC-content parameters, CpG-count constraints and exclusion motifs. Diamond-shaped sequences from each Pareto front were selected for further analysis based on codon similarity to ensure pool diversity. (B) Pairwise codon similarity (%) among WT, clinically used vaccines, vaccine candidates and newly designed sequences. CAI-based optimizations show high similarity to existing sequences with single-objective CAI optimization (CAI_Single) reaching 87.05% similarity to mRid and 84.46% to mRNA-1273. Sequences optimized under ICU–CC objectives show more uniform similarity (35.40–55.02%). (C) CpG site distribution across sequences.

## 3. Discussion

The rapid development of COVID-19 vaccines marked a major achievement in biomedical science and engineering. Nevertheless, the principles used to design their antigen-coding sequences remain insufficiently systematized. Thus, our analysis frames vaccine coding-sequence design as a multi-objective engineering problem in which codon usage, codon-pair context, GC content, CpG composition, regulatory motifs and RNA structural features interact in nonlinear and sometimes competing ways. Depending on the delivery platform, these features may affect transcription, RNA processing, innate immune recognition, transcript stability and translation at different stages of antigen production (figure 4). Consequently, vaccine sequence behaviour cannot be adequately represented by a single optimization metric. The proposed framework addresses this complexity by integrating multiple sequence-level criteria, adjustable sequence parameters and biological constraints into a common workflow for candidate generation and prioritization.

**Figure 4.**
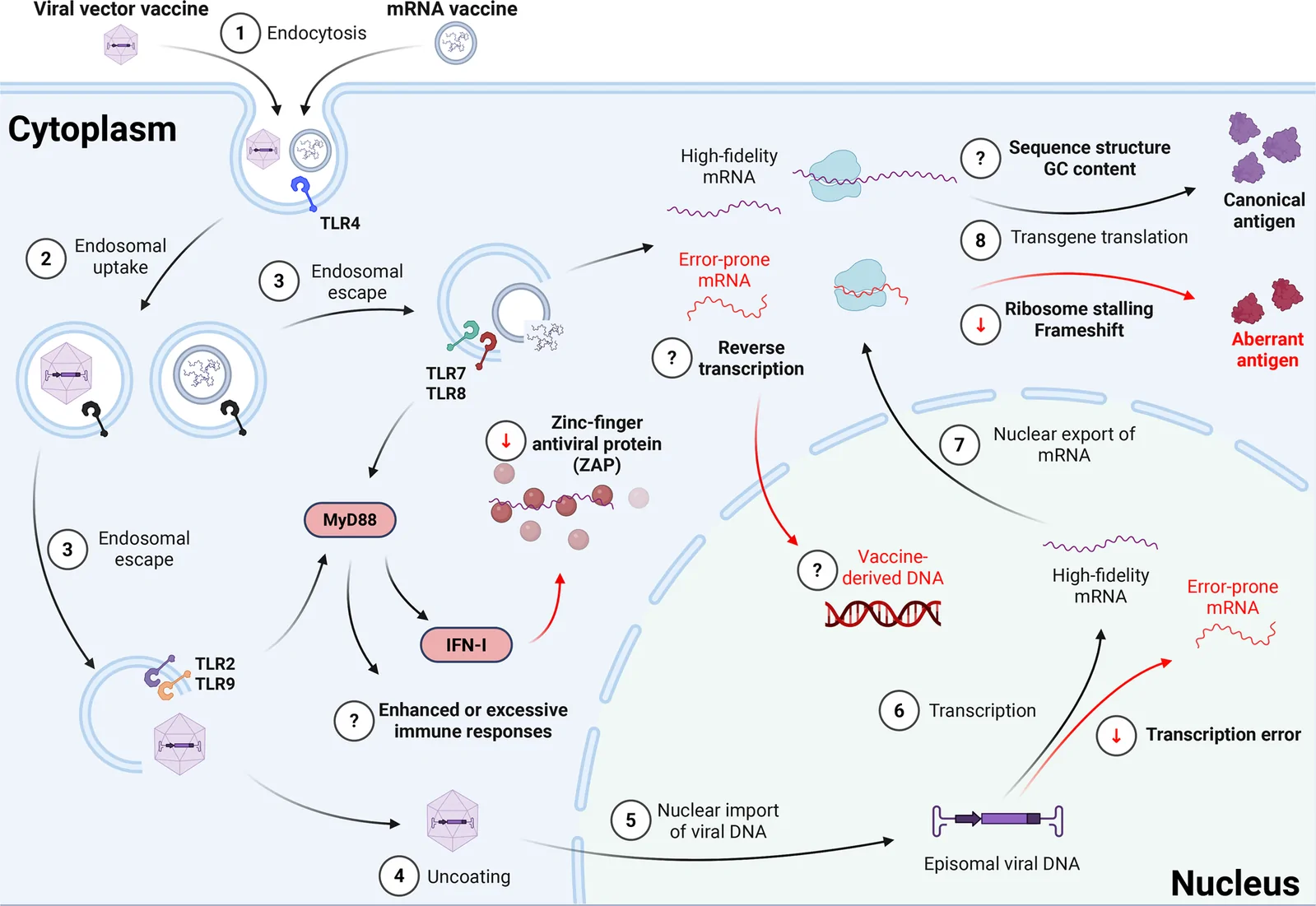
Conceptual map of sequence-sensitive checkpoints in viral vector and mRNA vaccine expression. Numbered circles indicate intracellular steps: (1) cell entry via endocytosis, (2) endosomal uptake, (3) endosomal escape, (4) capsid uncoating (viral vector only), (5) nuclear import of viral DNA, (6) nuclear transcription, (7) nuclear export of mRNA and (8) translation. Question marks denote steps with uncertain or conflicting experimental evidence, including downstream fates of vaccine-derived nucleic acids that remain outside the present scope and require further validation [62]. Stages with potential expression or sensing constraints are highlighted in red. At endosomal escape (step 3), ssRNA recognition by TLR7 and TLR8 may contribute to innate sensing modulated by nucleoside modification and purification strategy [63]. For viral vectors, capsid proteins can stimulate TLR2, and CpG motifs can activate TLR9, inducing cytokine and type-I interferon secretion via MyD88 [64]. For mRNA vaccines, LNP ionizable lipids have been reported to activate innate signalling, including TLR4-linked responses in some settings [59], potentially contributing to ZAP-related RNA restriction [58]. At nuclear steps (5–6; viral vectors only), cryptic splice sites and promoter-like elements may alter transcription. At translation (step 8), codon usage, codon-pair context, secondary structure, GC content and CpG abundance jointly shape ribosome behaviour with stronger local structure enhancing mRNA stability [48], but potentially slowing translation [65] or altering RNA-processing interactions [49,66]. Figure created using https://www.biorender.com/.

A central consideration is the choice of the codon-usage bias, which depends on the dataset used for its derivation. Potential sources include the tissue at the administration site, antigen-presenting cells such as dendritic cells [2,67], or broader human codon-usage datasets [68,69]. Accordingly, the same CAI metric can yield different values depending on the underlying codon profile [4,19,70]. In our analysis, clinically used vaccine sequences generally exhibited high CAI values when evaluated against CoCoPUTs, KAZUSA, Human Protein Atlas (HPA) and ribosomal-protein-derived profiles (supplementary material, table S4). Several tissues with limited direct relevance to vaccine administration also produced relatively high CAI values. These findings suggest that CAI mainly reflects codon preferences that are broadly shared across human reference sets and may have limited sensitivity to more subtle tissue-specific translational preferences.

CAI remains useful as a general measure of codon adaptation, but its interpretation should be complemented by additional descriptors, such as ICU and CC (figure 3A). BNT162b2 and mRNA-1273 both have high CAI values and provide broadly comparable protection against COVID-19 [71,72], although mRNA-1273 is administered at a higher dose [22] and elicits slightly higher IgG and IgA responses than BNT162b2 [14]. A recent study reported that specific proline codon pairs can strongly influence ribosome pausing and translation efficiency [73], further supporting that frequently used codon selection alone may be insufficient. The biological meaning of these metrics must, however, be interpreted within the relevant delivery platform. AZD1222 has CAI, ICU and CC values close to those of BNT162b2, although the two vaccines differ in reported efficacy [13] and protection [74,75] (supplementary material, table S5). Their coding sequences also share approximately 95% codon-level similarity. These observations demonstrate that sequence similarity and codon adaptation alone cannot account for product-level performance. In addition, vaccine efficacy is shaped by antigen configuration, dose, formulation, delivery efficiency, vector biology, pre-existing immunity and intracellular processing.

RNA structure introduces a trade-off between stability and translation [4]. Reduced folding near the translation initiation site generally facilitates ribosome access [76], whereas moderate structure elsewhere may protect transcripts from degradation [48]. Excessive or unusually stable folding can instead impede elongation, induce ribosome stalling or accelerate RNA decay [65,66,77]. Guanine-rich regions, for example, can form four-stranded G-quadruplexes that may obstruct transcript elongation and reduce mRNA stability [49]. mRid and mRNA-1273 were closely matched at the codon level and had nearly identical GC contents and high CAI values. However, G4Hunter analysis under default parameters predicted 16 quadruplex-propensity hits in mRid and only nine in mRNA-1273 [78]. Thus, regional assessment of G-quadruplexes, pseudoknots, initiation-site accessibility and other localized structural features may provide information that is not captured by conventional codon or global folding metrics.

Several limitations of the current framework should be considered when analysing the present results. First, the framework supports computational characterization and candidate prioritization but does not directly predict antigen expression, immunogenicity or clinical efficacy. The functional effects of codon patterns, CpG configurations, cryptic motifs and predicted structures require experimental validation. Clinical comparisons are also confounded by differences in formulation, dose, dosing interval, study population, circulating variant and trial design. Second, full-length RNA folding was not incorporated because prediction reliability decreases and computational cost increases with sequence length [79], and pseudoknots were not predicted for the same reason [80]. Standard thermodynamic models also do not adequately account for modified nucleotides such as N1-methylpseudouridine [81]. Third, the current analysis focused mainly on coding regions and excluded UTRs, poly(A) tails, cap structures and other non-coding elements that strongly affect translation and transcript stability [2]. Finally, tissue-specific codon references were derived primarily from transcript abundance, which does not directly represent ribosome occupancy, tRNA availability or translational efficiency [82,83].

In the study, we proposed an integrated multi-parameter strategy, which can balance innate immune sensing, translation and sequence stability in genetic vaccine design. Although demonstrated using the COVID-19 spike protein, the framework can be extended to antigens of varying types and lengths, including multi-antigen constructs relevant to future outbreaks. Such applications may expand the search space and increase computational cost. The framework could also be coupled with protein structure and stability prediction, linking coding-sequence design to the folding and expression of the encoded antigen. Future development could incorporate regional RNA-folding metrics, G-quadruplex and pseudoknot predictions, modified-nucleotide models and non-coding sequence optimization. Ribosome-profiling, proteomic and tRNA-abundance data may improve context-specific codon references. Potentially, machine-learning models trained on matched sequence, expression, innate-sensing and immunogenicity data could further prioritize candidates from larger Pareto fronts. Such extensions would support application to longer antigens, multi-antigen constructs, different delivery platforms and host populations varying in age, sex, genetic background, health status and pre-existing immunity [40–42,84]. Overall, this study establishes genetic vaccine coding-sequence design as a context-dependent, multi-objective problem, providing a rational basis for comparing existing sequences and prioritizing new candidates with explicitly defined biological trade-offs.

## 4. Material and methods

### 4.1. Sequence codon similarity

Sequence codon similarity was computed by comparing each pair of sequences and counting the number of matching codons at corresponding positions. For each comparison, sequences were aligned from the start codon. A match between codons was assigned a value of 1, while a mismatch was assigned a value of 0. The pairwise similarity percentage was calculated by summing these values, dividing by the total number of codons and multiplying by 100. When sequence lengths differed, the similarity percentage was normalized by the length of the left-hand sequence in the heat map matrix.

### 4.2. Codon usage bias establishment

To establish codon reference bias for human tissues, we retrieved the ‘RNA consensus tissue gene data’ from the HPA [85] database, which includes transcriptomic profiles across 54 different tissues. Additionally, we used the ‘RNA HPA immune cell gene data’ to obtain expression profiles specific to myeloid dendritic cells. During pre-processing, genes annotated as mitochondrial genes, ribosomal genes, retired genes and pseudogenes were filtered out using NCBI's RefSeq database annotations. From the remaining genes, we selected the top 1% exhibiting the highest expression levels based on descending nTPM (normalized transcripts per million) values, a metric representing transcript abundance of protein-coding genes across samples in the HPA. In addition, we separately generated codon bias profiles derived primarily from human nuclear-encoded ribosomal protein gene sets, which are well known for their high expression levels. For broader comparative purposes, we also incorporated genome-wide codon usage frequency data from the KAZUSA database [68], which lacks codon pair information, as well as both codon and codon-pair usage data from the CoCoPUTs database [69].

### 4.3. Codon index calculations

The CAI quantifies the degree of bias towards codons that are known to be preferred in reference gene sets, with a range of 1 representing maximum bias to 0 indicating minimum bias [43]. To compute the CAI, one must first calculate the relative synonymous codon usage (RSCU) value for each codon. This value is determined by dividing the observed number of codon occurrences by the expected number, assuming that all synonymous codons for a given amino acid are used with equal frequency. The formula for calculating the RSCU value for synonymous codon ‘*i*’ of an amino acid that is ‘*n*-fold degenerate’ is as follows:

$$\;RSCU_{i}=\;\frac{X_{i}}{\frac{1}{n}\sum_{i\;=\;1}^{n}X_{i}},$$

where *X_i_* is the number of occurrences of codon *i* and *n* is 1, 2, 3, 4, or 6.

Analogously, a ‘relative adaptiveness’ value, *w_i_*, for codon *i* is calculated from its relative frequency of use in a reference set of genes, which may consist of either highly expressed or condition-specific genes:

$$w_{i}=\frac{RSCU_{i}}{RSCU_{max}}.$$

Here, RSCU_max_ denotes the RSCU of the most frequently used synonymous codon for that amino acid. The CAI for a gene is then defined as the geometric mean of *w* values for codons in that gene, where *L* is the number of codons in the gene:

$$CAI={\left(\overset{L}{\underset{i=1}{\prod }}w_{i}\right)}^{1/L}.$$

The ICU is represented as a vector whose components are the frequencies of individual codons [44]. Each codon's frequency is computed by dividing its count in a coding sequence by the total count of the corresponding amino acid in the target protein sequence. The ICU distribution is a 64-element vector analogous to the RSCU vector, except that it omits the 1/*n* term in the denominator. Consequently, the ICU fitness of a subject in relation to the reference can be articulated as the negative of the Manhattan distance between the reference frequency (*p*_0_) and the frequency of the gene of interest (*p*_1_):

$$\Psi_{ICU}=-\frac{\left\|p_{0}-p_{1}\right\|}{64}=-\frac{\sum_{i=1}^{64}\left|p_{0}^{i}-p_{1}^{i}\right|}{64}.$$

The calculation for CC is similar to that for ICU, with the only difference being that it accounts for pairs of codons. The number of possible codon pair combinations is 3904 out of 4096, as stop codons cannot occupy the leading position in a codon pair. In the same way, the CC fitness of a subject in relation to the reference can be articulated as the negative of the Manhattan distance between the reference frequency (*q*_0_) and the frequency of the gene of interest (*q*_1_):

$$\Psi_{CC}=-\frac{\left\|q_{0}-q_{1}\right\|}{3904}=-\frac{\sum_{i=1}^{3904}\left|q_{0}^{i}-q_{1}^{i}\right|}{3904}.$$

### 4.4. Sequence design and codon optimization

New 2P-stabilized spike vaccine candidate sequences were designed using a previously developed codon optimization method [44]. We selected skeletal muscle as the codon-bias reference because it is the site of vaccine administration [13] and is involved in local immune signalling pathways, including cytokine and chemokine expression [45]. We conducted codon optimization under two primary scenarios using CoDOn [86]. Scenario 1 used CAI as the sole objective function. Scenario 2 employed multi-objective optimization considering ICU and CC. The CAI scenario was further divided into a minimal design (CAI only) and an extended design with additional sequence-level constraints and parameters allowing trade-offs among competing parameters. To refine the search, we applied additional criteria, including maintaining GC content between 30 and 70% and excluding the cryptic promoter and splice-site motifs defined in the main text. In this scenario, the number of CpG motifs was set to 239, corresponding to the expected value for viral or bacterial genomes under CAI optimization with additional parameters and sequence constraints. For ICU–CC optimization, the CpG motif count was systematically varied, starting from 0 and increasing in increments of 29 up to 290, based on the 29 motifs present in the WT. For comparison, an additional case was included where all other settings were identical but without applying any constraint on the CpG count. The optimization process primarily targeted maximizing CAI or ICU and CC fitness while satisfying these parameters and constraints. Owing to the complexity and nonlinearity of CC optimization, genetic algorithms were employed to obtain feasible solutions within a reasonable computational timeframe. The search was programmed to terminate when the fitness change remained below 0.5% over 100 iterations or upon reaching 150 000 iterations. After obtaining each Pareto front, eight solutions were selected such that the distances between them were approximately uniform. From each front, one sequence was then selected for additional analyses by prioritizing lower codon similarity when compared with clinically used vaccines, vaccine candidates and the WT, in order to maximize the diversity of the sequence pool.

## Supplementary Material



## Data Availability

Scripts for the descriptive analyses, including codon similarity, codon-usage metrics, motif screening and CpG profiling, are available in Zenodo [87]. All optimization objectives, parameter settings, selected candidate sequences and objective-score tables used in this study are provided in full detail within the paper and the supplementary material to ensure reproducibility of the reported analyses. Supplementary material is available online [88].
